# Longitudinal analysis of microcirculatory parameters in gingival tissues after tooth extraction in patients with different risk profiles for wound healing disorders – a pilot study

**DOI:** 10.1007/s00784-024-05686-3

**Published:** 2024-05-08

**Authors:** Alexandra Mayr, Nadja Ciper, Gerhard Wahl, Jan Wildenhof, Stilla Frede, Christian Kirschneck, Andreas Jäger, Werner Götz, Svenja Beisel-Memmert

**Affiliations:** 1 Department of Orthodontics, University Hospital Bonn, Medical Faculty, Bonn, Germany; 2 Center for Dental, Oral and Maxillofacial Medicine, University Hospital Bonn, Medical Faculty, Bonn, Germany; 3 Private Clinic Schloss Schellenstein, Olsberg, Germany; 4 Department of Anaesthesiology, University Hospital Bonn, Medical Faculty, Bonn, Germany

**Keywords:** Wound healing disorder, Tooth extraction, Periodontitis, Microcirculation, Spectrophotometry

## Abstract

**Objectives:**

We aimed to establish a risk profile for intraoral wound healing disorders based on measurements of microcirculation in gingival tissues.

**Materials and methods:**

Oxygen saturation (SO_2)_ and blood flow in gingival tissues were measured with tissue spectrometry and laser doppler spectroscopy in 37 patients before/after tooth extractions. Patients were assigned to four groups: anamnestically and periodontally healthy patients (*n* = 7), anamnestically healthy but suffering from periodontitis (*n* = 10), anamnestically healthy but smoking and suffering from periodontitis (*n* = 10) and suffering from diabetes and periodontitis (*n* = 10). Measurements were performed at three different time points: Baseline measurement (T0), one day *post extractionem* (p.e.) (T1) and seven days p.e. (T2).

**Results:**

Baseline SO_2_ values were higher in control patients (*p* = .038). This effect was most evident in comparison to smokers suffering from periodontitis (*p* = .042), followed by diabetics suffering from periodontitis (*p* = .09). An opposite trend was seen for blood flow. Patients suffering from periodontitis demonstrated higher blood flow values (*p* = .012). Five patients, which belonged to the group of smokers suffering from periodontitis, showed clinically a delayed wound healing.

**Conclusion:**

Differences in SO_2_ and blood flow of gingival tissue could be detected in different groups of patients with existing periodontitis compared to control patients.

**Clinical Relevance:**

Lower baseline SO_2_ values could be a warning signal for possible wound healing disorders after oral surgery.

## Introduction

Periodontitis is the most common reason for tooth loss in Germany for patients over 40 years of age [1]. The Fifth German Oral Health Study (DMS 5) showed that more than half of the adult population suffers from either moderate or severe periodontitis. This widespread disease is of a multifactorial etiology. It is caused by periodontal pathogenic microorganisms and influenced by genetic and environmental factors [2, 3]. Significant periodontal risk factors identified in the DMS 5 include smoking and diabetes mellitus [1, 3].

If teeth are no longer worth preserving due to advanced bone loss and periodontal inflammation, they are usually extracted. An extraction wound presents a complex wound with simultaneous soft tissue and bone involvement. A wound healing disorder in the area of extraction wounds can be caused by bone impingement during extraction, by extractions in an acute inflammatory stage, e.g. in the presence of periodontitis, as well as by dry alveoli, e.g. as a result of smoking [4, 5]. A number of metabolic diseases, such as diabetes mellitus, can also lead to the occurrence of wound healing disorders post extraction [4, 6].

In 2003, Manassa et al. were able to show in a clinical study that smokers develop wound healing disorders three times more often than non-smokers [7]. This is due, among other things, to a microcirculatory disturbance caused by nicotine consumption, as this causes a local release of noradrenaline and adrenaline, and thus a vasoconstriction of the peripheral blood vessels [8, 9]. Smokers are experiencing a reduction in oxygen transport and metabolism. This in turn results in a hypoxic environment, which can lead to impaired wound healing [8, 10, 11].

Patients suffering from diabetes mellitus also show impaired wound healing in the context of surgery. This is often attributed to a malfunction of polymorphnuclear leukocytes and macrophages as well as a disturbance in the production of growth factors [12, 13]. A development of microangiopathies in the presence of diabetes mellitus also seems to play an important role. In the small blood vessels, changes and thickening of the basement membrane occur, which can cause stenosis or even occlusion of the blood vessels concerned, resulting in a reduced oxygen supply to the surrounding tissues [14, 15].

Microcirculatory disturbances in general are an important component of various disease processes and contribute significantly to wound healing disorders. Both tissue perfusion and oxygenation are essential cornerstones of physiological wound healing. The search for an optimal procedure to monitor microcirculation is of particular interest in many clinical settings [16–20]. With the help of a good monitoring process, wound healing disorders could be detected at an early stage before they manifest clinically. By doing so, possible complications could be treated in time or may even be avoided. First steps have already been taken to enable the clinician to monitor perfusion and oxygenation in oral tissues with a non-invasive and effective method. Barry and colleagues used the O2C device (Oxygen to See, LEA Medizintechnik, Gießen, Germany) which combines white light spectrometry and laser Doppler spectroscopy to measure perfusion parameters in healthy adults [21]. Another study used the same device to monitor oral tissue flaps during and post-surgery to avoid healing disorders and flap failure emphasizing the role of perfusion and oxygenation during wound healing [22].

Aim of this pilot-study was to determine, whether it is possible to establish a risk profile for intraoral wound healing disorders based on measurement parameters of the microcirculation in the area of the gingival tissue. The following hypotheses were investigated: (1) There are no differences in oxygen saturation (SO_2_ in %) and blood flow (Flow in AU = Arbitrary Unit) of gingival tissue in different groups of patients with existing periodontitis compared to control patients. (2) There is no difference in the extent of change in oxygen saturation (SO_2_) and blood flow measured in the course of wound healing after tooth extraction depending on a patients smoking behaviour, periodontal health and diabetes status.

## Subjects and methods

### Study design and sample

This investigation was planned as a pilot-study and data primary used under other questions as basis for a dissertation [23]. It was the aim to test the intraoral applicability of the measuring probe head on the marginal gingiva and to determine first quantitative data for the parameters (means and standard deviations) of the microcirculation in the area of the gingival tissue following tooth extractions in order to perform a sample size calculation for a future larger trial. For this purpose, as a general rule of thumb, Browne [24] and Sim and Lewis [25] recommended a sample size between 30 and 50.

Thus, 37 patients (20 males and 17 females) aged 14–77 with a mean age of 53 undergoing tooth extraction were included in the study. Sample inclusion criteria for the experimental group A (EG A) included patients (1) suffering from periodontitis, (2) who were otherwise anamnestically healthy and (3) non-smoking (*n* = 10). Experimental group B (EG B) consisted of patients (1) suffering from periodontitis, (2) smoking and (3) otherwise anamnestically healthy (*n* = 10). Experimental group C (EG C) consisted of patients (1) suffering from periodontitis, (2) suffering from diabetes and (3) were non-smoking (*n* = 10). The control group (CG) comprised seven anamnestically and periodontally healthy, non-smoking subjects.

The exclusion criteria for all groups were (1) diseases relevant to the patient’s medical history, which required medication that altered coagulation or blood flow, (2) severe obesity and (3) antihypertensive therapy or hypertonia. In order not to distort the results due to individual characteristics, the patients were only included in the study once with one tooth extraction each. For each extracted tooth, severity of gingivitis or periodontitis was recorded prior to the surgical procedure. Probing depths as well as degree of tooth mobility and bleeding on probing were determined. The main reason for tooth extraction was that the tooth was considered as no longer worth preserving for periodontal reasons by specialists from the Clinic of Dental Medicine in the periodontitis groups. In the control group however the tooth was extracted for orthodontic reasons.

Informed written consent was obtained from all patients. This prospective study was reviewed and approved by the Ethics Committee of the Medical Faculty of the University of Bonn (protocol no. 086/11).

### Surgical procedure and data acquisition

**Fig. 1:**
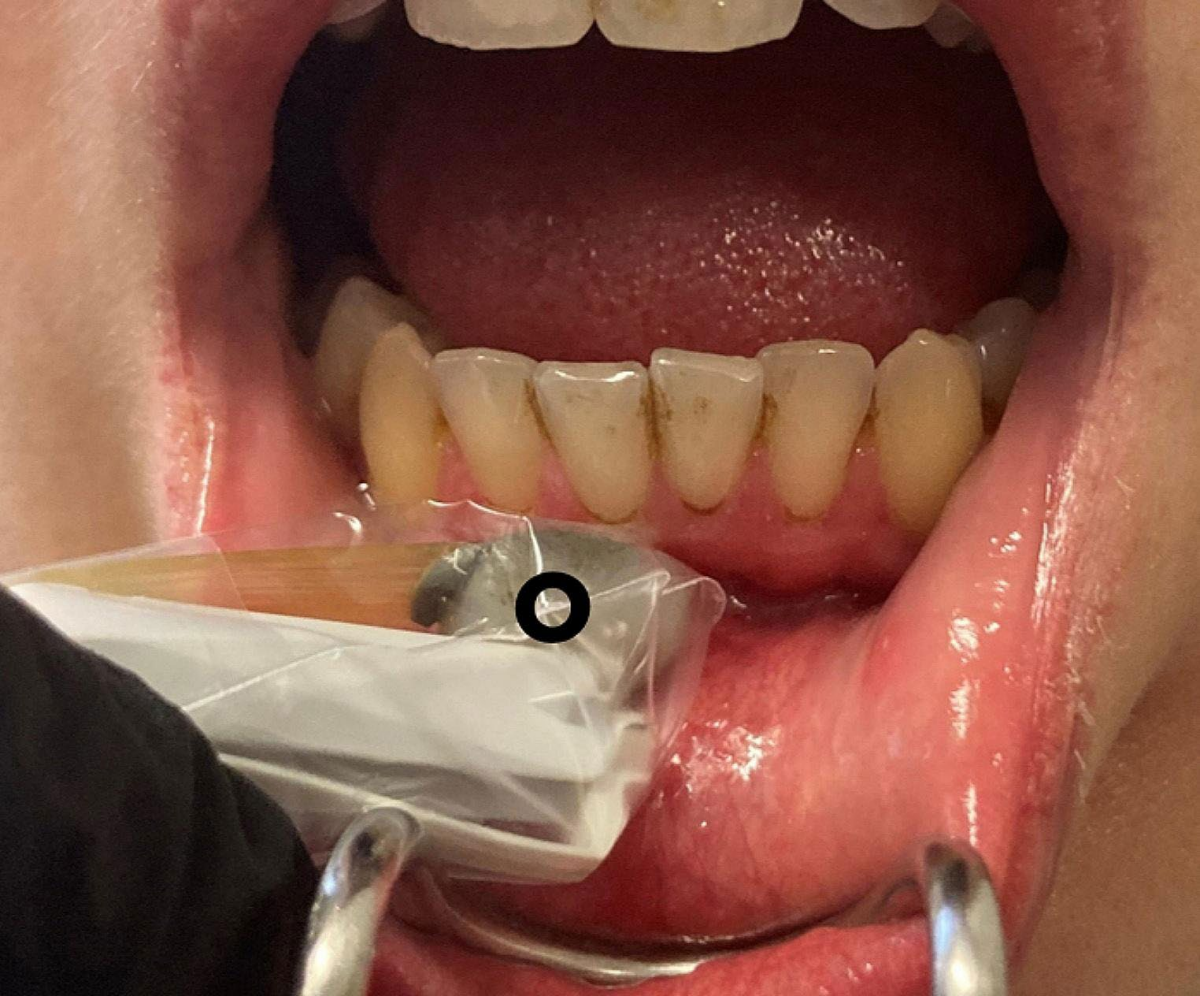
Intraoral positioning of the measuring probe O2C (Oxygen to See, LEA Medizintechnik, Gießen, Germany). The flexible head of the measuring probe is indicated by circle (°)

In order to produce valid values under standardized conditions, as described in the literature, care was taken with each patient to include a 15-minute resting phase before the start of the measurements so that the blood pressure could settle at a constant level [26]. Furthermore, since both cold and pain can lead to peripheral vasoconstriction, a comfortable temperature as well as freedom from pain during the measurements were ensured [27, 28]. The measurements were performed with a type LF-2 probe of the micro-lightguide spectrophotometer O2C (Oxygen to See, LEA Medizintechnik, Gießen, Germany; see Fig. 1), a non-invasive device which is used to determine SO_2_ and blood flow in clinical settings, in a transparent probe sheath [29]. Tissue spectrometry can be affected by bright light, because these extraneous lights interfere with the reflected light of the hemoglobin and thus lead to false readings of SO_2_. To avoid this in the present study, the room was darkened by curtains and the surgical light was switched off. Since Laser Doppler spectroscopy measurements may be sensitive to motion artifacts the patient was asked to avoid any movement during the examination and the surgeon kept his hand as still as possible during the measurements.

It has already been shown in previous studies that this non-invasive technique to monitor SO_2_ and blood flow does not only work on the epidermis, for example to monitor the healing of skin grafts, but also on the oral mucosa [21, 30].

The measurements were taken on the vestibular side in the area beyond the mucogingival junction, the alveolar mucosa, of the tooth to be extracted. Another measuring point was located vestibular in the alveolar mucosa of the contralateral tooth, which was not to be extracted (= control tooth). If the extraction instructions included the extraction of another tooth, measurements were also taken there. The measuring probe (type LF-2) was placed with a continuous contact pressure of 0.25 N on each area of interest and data for two parameters were recorded: SO_2_ and relative blood flow.

Measurements were performed at three different time points: Baseline measurement before local anaesthesia application (T0), measurement one day *post extractionem* (p.e.) (T1) and measurement seven days p.e. immediately before suture removal (T2). Each measurement consisted of three individual measurements per tooth in order to subsequently calculate the mean value. For the assessment of differences (Δ) between the mean oxygen and blood flow readings before tooth extraction and one (T1) respectively seven (T2) days after tooth extraction, ΔSO_2_ was determined by calculating the difference of the measured oxygen saturation before local anesthesia minus the measured oxygen saturation one respectively seven days p.e. (T1 – T0, T2 – T0, respectively). ΔFlow was calculated analogously to ΔSO_2_. In addition, blood pressure, pulse and oxygen saturation of the patient were recorded at each time point. All measurements were performed by the same clinician.

### Statistical analysis

Results are expressed as mean ± standard error of the mean. Statistical analyses were performed using SPSS software (SPSS Inc., Chicago, Illinois). Statistical differences in the measured data were calculated using ANOVA. Two-sample comparisons were performed using *t*‐test. A *p* value < 0.05 was considered statistically significant and indicated by asterisk (*) in the figures. Effect sizes were calculated based on Cohen’s d, with d ≥ 0.2 = small effect, d ≥ 0.5 = medium effect, and d ≥ 0.8 = large effect.

## Results

First, we investigated whether there were differences in mean oxygen readings SO_2_ (%) and blood flow (AU) before tooth extraction for the patients presenting with a different risk status (healthy, periodontitis, periodontitis + smoking, periodontitis + diabetes). Analysis of our clinical findings showed the following results (see Table 1). Comparisons of the groups were conducted using the t-Test (see Table 2). Our clinical findings showed that the (mean) oxygen saturation values SO_2_ (%) before tooth extraction proved to be statistically significantly higher in the control patients (78.86 ± 9.87) compared to the patients suffering from periodontitis (73.13 ± 6.81, *p* = .038). This effect was most evident when comparing the control patients to the smokers suffering from periodontitis (71.00 ± 3.65, 72.20 ± 9.39, *p* = .042), followed by the diabetics suffering from periodontitis (*p* = .09) and least pronounced when comparing the control patients to the anamnestically healthy subjects but suffering from periodontitis (76.20 ± 5.61, *p* = .245). Values for mean blood flow (AU) before tooth extraction for the different risk groups appeared statistically lower in the control patients (250.14 ± 62.42) compared to the patients suffering from periodontitis (317.67 ± 110.05, *p* = .065). This effect, indicating an increase in reactive vascularization, exclusively showed statistical significance when comparing the control patients to the anamnestically healthy subjects but suffering from periodontitis (348.50 ± 5.61, *p* = .012). In contrast, smoking (312.60 ± 116.29, *p* = .109) or diabetes mellitus (291.90 ± 125.67, *p* = .191) in addition to periodontitis only caused a moderate increase in blood flow.

**Table 1:** Distribution and mean SO_2_/Flow by risk status before tooth extraction

Risk status	n	mean SO_2_ (%)	Flow (AU)
Healthy (control group)	7	78.86 ± 9.87	250.14 ± 62.42
Periodontitis in total (EG A + EG B + EG C)	30	73.13 ± 6.81	317.67 ± 11.42
Periodontitis only (EG A)	10	76.20 ± 5.61	348.50 ± 5.61
Periodontitis only (EG B)	10	71.00 ± 3.65	312.60 ± 116.29
Periodontitis + diabetes (EG C)	10	72.20 ± 9.39	291.90 ± 125.67

**Table 2:** Differences (t-test) for (A) SO_2_and (B) Flow values before tooth extraction with relative significances (*p*) and effect size (*d*)

A) SO_2_ - Oxygen saturation	*p*	d
Healthy vs. periodontitis in total	0.038 *	0.79
vs. periodontitis only	0.245 n.s.	0.37
vs. periodontitis?+?smoking	0.042 *	1.22
vs. periodontitis?+?diabetes	0.09 n.s.	1.03

B) Blood flow		
Healthy vs. periodontitis in total	0.065 n.s.	0.67
vs. periodontitis only	0.012 *	1.32
vs. periodontitis?+?smoking	0.109 n.s.	0.68
vs. periodontitis?+?diabetes	0.191 n.s.	0.42

Next, we examined whether there were variations in the difference (Δ) between the mean oxygen readings SO_2_ and blood flow before tooth extraction and the mean oxygen readings SO_2_ and Flow one (T1) respectively seven days (T2) after tooth extraction for the different risk groups (healthy, periodontitis, smoker, diabetes) (see Table 3). Further analysis was conducted using t-Test (see Table 4). Our subsequent analysis regarding the extent of change in SO_2_ and blood flow measured in the course of wound healing after tooth extraction showed, that there was no noticeable difference according to the patient’s risk status.

**Table 3:** Differences (Δ) in the mean value of SO_2_ and Flow by risk status one (T1) respectively seven days (T2) after tooth extraction. ΔSO_2_was determined by calculating the difference of the measured oxygen saturation before local anesthesia (T0) minus the measured oxygen saturation one (T1) respectively seven days (T2) p.e. ΔFlow was calculated analogously to ΔSO_2_

Risk status	*n*	ΔSO_2_(%) (T1 - T0)	ΔSO_2_(%) (T2 - T0)
Healthy (control group) periodontitis in total (EG A+EG B+EG C)	7 30	1.86 ± 11.63 0.40 ± 9.53	5.43 ± 12.53 -0.43 ± 8.15

Risk status	n	ΔFlow (AU) (T1 ? T0)	ΔFlow (AU) (T2 ? T0)
Healthy (control group) periodontitis in total (EG A+EG B+EG C)	7 30	-114.71 ± 81.27 -62.17 ± 143.68	-46.86 ± 92.53 -19.90 ± 128.44

**Table 4:** Differences (t-test) for ΔSO_2_and ΔFlow values with relative significances (p) and effect size (d)

A) ΔSO_2_(%)	(T1 - T0)	(T2 - T0)	
Healthy vs. periodontitis in total	0.364 n.s.	0.066 n.s.	*p*
	0.15	0.67	*d*

B) ΔFlow (AU)	(T1 - T0)	(T2 - T0)	
Healthy vs. periodontitis in total	0.18 n.s.	0.303 n.s.	*p*
	0.40	0.23	*d*

Clinically, no patient treated in the course of this study developed a wound healing disorder after tooth extraction. Only five patients, from EG B (smokers with periodontitis), presented with a slight delay in wound healing.

## Discussion

The aim of this study was to determine whether it is possible to establish a risk profile for intraoral wound healing disorders based on measured parameters of the microcirculation in the surrounding gingival tissue of the extraction socket.

The results of the current study show that the values for oxygen saturation in healthy patients in the initial situation were significantly higher than in patients suffering from periodontitis. When differentiating the results according to varying expressions of the risk status it was found, that smokers with periodontitis had the lowest baseline oxygen saturation values at the experimental area of tooth extraction, followed by diabetics with periodontitis. Interestingly, smokers with periodontitis were the only patients in whom also delayed wound healing was observed. An opposite trend as in oxygen saturation was seen for blood flow. Patients with periodontitis had statistically significantly higher blood flow values than healthy patients. When determining the extent of change in oxygen saturation SO_2_ and blood flow measured in the course of wound healing after tooth extraction, it could be seen that there was no noticeable difference according to the patient’s health status.

In general, wound healing leads to an increased oxygen demand and consumption due to oxygen-dependent processes of the cellular components of the immune system [8, 31, 32]. Furthermore, in the course of wound healing, vasodilation occurs in the exudative phase during the first eight hours followed by an increase in microcirculation. Subsequently, neoangiogenesis takes place in the resorptive phase over one to four days, which leads to an increase in blood flow in the wound environment [15, 31, 33].

Our results show that the (mean) oxygen saturation values SO_2_ (%) before tooth extraction were statistically significantly higher for the control patients than for the patients with periodontitis. The observed differences were most pronounced when the control patients were compared to smokers with periodontitis, followed by diabetics with periodontitis. The differences in oxygen saturation were least pronounced when comparing control patients to individuals with a healthy medical history but suffered from periodontitis. These results are in line with the relevant literature. As early as 1988, Shizukuishi et al. used tissue spectrometry to show that gingival tissue in patients suffering from periodontitis has a lower oxygen saturation [34]. Smokers exhibit limitation in oxygen transport and metabolism, and a hypoxic environment develops, which can lead to wound healing disorders [8, 10, 11]. Furthermore, smoking leads to a constriction of the blood vessels, resulting in poorer blood circulation throughout the body and thus also in the oral cavity [35–38]. Nicotine leads to plaque formation and stenosis in the blood vessels and exhibits a vasoconstrictory effect, leading to a decrease of gingival blood flow in the oral cavity [39, 40]. In addition, tissue vascularization is significantly reduced. Mirbod et al. found a significant change in the volume of blood vessels in smokers. As a result, fewer large blood vessels could be detected in the tissues of smokers, but more small ones. Clinically, this resulted in a reduced bleeding on probing [41–43]. Another reason why patients with cigarette consumption or diabetes mellitus in addition to periodontitis present with even lower oxygen saturation values could be the development of a hypoxic state due to the high metabolic demand of migrating immune cells in the context of periodontitis [44]. Additionally, rapidly proliferating microorganisms contribute to hypoxia as they quickly consume the available oxygen [45]. Smoking and diabetes mellitus have been shown to further increase the tissue hypoxia resulting from periodontitis [8].

In this study, blood flow (AU) before tooth extraction was lower in the control patients than in the patients with periodontitis. However, the values only reached statistical significance when the control patients were compared to the periodontitis patients, which were otherwise healthy. Additional cigarette consumption or diabetes mellitus caused blood flow to be altered only in a non-statistically relevant range. These results can be well classified and explained based on the existing literature. Due to the inflammation present in periodontitis, the immune system provides sufficient defense cells to the site by dilating the blood vessels and consequently increasing blood flow [46, 47]. However, in case of cigarette consumption or in diabetes mellitus, vasoconstriction is induced by fibrosis and sclerosis of the vessel walls [36, 37, 39, 48, 49]. The results available in this study probably provide evidence that an existing periodontitis appears to exert a greater effect on blood flow than existing nicotine consumption or diabetes mellitus does.

The next question to be addressed was whether a change in oxygen saturation and blood flow in the gingival tissue of the different groups of patients with existing periodontitis had a negative effect on wound healing following tooth extraction. Although we could show that there were statistically significant differences in (mean) oxygen readings SO_2_ (%) and Flow (AU) before tooth extraction for the different risk groups, our subsequent analysis regarding the change in SO_2_ and blood flow measured in the course of wound healing after tooth extraction showed that there was no noticeable difference according to the patient’s risk status. Nevertheless, a slightly delayed wound healing was observed in five patients. All five patients were from the group of smokers suffering from periodontitis, which also had statistically significant lower baseline oxygen saturation values. In the future, low baseline oxygen saturation values before local anesthesia might be used as a warning signal for possible wound healing disorders after oral surgery. This has to be investigated in further studies. In the field of oncology, as well as in radiology, it has already been successfully shown that monitoring tissue oxygen saturation can help to identify patients with an increased risk of wound healing disorders at an early stage [50, 51].

In a clinical setting monitoring wound of healing disorders, procedures that are non-invasive, reliable, easy chairside to use providing reproducible and objective results are of great importance to allow early detection before clinical manifestation. Various methods have been applied, which can be divided into two major groups. Monitoring methods for tissue perfusion on the one hand and measuring methods for the determination of tissue oxygenation on the other hand.

Microcirculation in the oral mucosa was analyzed by tissue spectrometry and laser Doppler spectroscopy, which was shown to be reproducible and reliable [34, 52–57]. Although tissue spectrometry is acknowledged to be a reliable and sensitive monitoring method, its susceptibility to interference from extraneous light has been considered a disadvantage. At the same time, Laser Doppler spectroscopy was mostly considered feasible, but the high susceptibility to motion artifacts as well as the lack of absolute values, and therefore the consequent difficulty in interpreting and comparing measurement results, have been criticized. The O2C instrument from LEA (Giessen, Germany) used in this study combines the two methods in a single instrument and has been shown to produce reproducible and reliable results on both the oral mucosa and the skin [21, 30]. Forst et al., tested its reliability of the O2C instrument for the assessment of skin microvascular function during the postischemic reactive hyperemia response brought on by suprasystolic occlusion via a cuff on the upper arm and found it to be an easy, noninvasive, and reliable method for simultaneous measurement of superficial microvascular blood flow by laser Doppler fluxmetry and skin oxygenation by spectrophotometry [58]. Despite its non-invasiveness and ease of use, the disadvantage of the Laser Doppler spectroscopy remains that, apart from the SO_2_ parameters, only relative values, which can only be interpreted to a limited extent, are produced. In this study, we included the measurements of the contralateral control tooth to take variation of the perfusion parameters between the maxilla and the mandible into account. However, it is also important to ensure that possible interfering factors are excluded with this technique. In order to exclude movement artifacts, at least from the surgeon, and to enable reproducible measurements on different measurement days, the use of a patient-specific splint would have been advantageous. This was already established in numerous studies, which solely used laser Doppler spectroscopy [59, 60]. However, this was not possible in the present study, because the use of a splint is always combined with a certain amount of pressure on the probe and on the tissue. With the practical knowledge of this study, it is planned to design a new type of probe for future studies, which will allow the pressure-free application in combination with the use of a patient-specific splint. In order to avoid cross-contamination, a sterile transparent probe cover was used. In a preliminary trial of the study, no differences in skin measurements with and without a transparent probe sheath were identified.

## Conclusion

Differences in SO_2_ and blood flow of gingival tissue could be seen in different groups of patients with existing periodontitis compared to control patients. Lower baseline oxygen saturation values may be a warning signal for possible wound healing disorders after oral surgery, which can be detected reliably and non-invasively with the O2C instrument from LEA. However, the measuring probes currently available are susceptible to disturbance variables. Therefore, in the further development of the device, emphasis should be placed on optimizing the probe for intraoral measurements. At the end of the pilot-study, a new probe design was submitted with the request for implementation to the manufacturer (LEA, Gießen, Germany) for subsequent planned clinical studies. In the future, this should make it possible to apply the probe with the aid of a patient-specific splint without having to apply pressure to the sensible tissues.

## Data Availability

No datasets were generated or analysed during the current study.

## References

[1] Jordan RA, Bodechtel C, Hertrampf K, Hoffmann T, Kocher T, Nitschke I (2014). The fifth German oral health study (Funfte Deutsche Mundgesundheitsstudie, DMS V) - rationale, design, and methods. BMC Oral Health.

[2] Fragkioudakis I, Riggio MP, Apatzidou DA (2021) Understanding the microbial components of periodontal diseases and periodontal treatment-induced microbiological shifts. J Med Microbiol 70(1). 10.1099/jmm.0.001247 10.1099/jmm.0.001247 33295858

[3] Hajishengallis G, Chavakis T, Lambris JD (2020). Current understanding of periodontal disease pathogenesis and targets for host-modulation therapy. Periodontol 2000.

[4] Akinbami BO, Godspower T (2014). Dry socket: incidence, clinical features, and predisposing factors. Int J Dent.

[5] Kusnierek W, Brzezinska K, Nijakowski K, Surdacka A (2022) Smoking as a risk factor for dry socket: a systematic review. Dent J (Basel) 10(7). 10.3390/dj10070121 10.3390/dj10070121 PMC9317683 35877395

[6] Zhang S, Song S, Wang S, Duan Y, Zhu W, Song Y (2019). Type 2 diabetes affects postextraction socket healing and influences first-stage implant surgery: a study based on clinical and animal evidence. Clin Implant Dent Relat Res.

[7] Manassa EH, Hertl CH, Olbrisch RR (2003). Wound healing problems in smokers and nonsmokers after 132 abdominoplasties. Plast Reconstr Surg.

[8] Guo S, Dipietro LA (2010). Factors affecting wound healing. J Dent Res.

[9] Silva H (2021) Tobacco Use and Periodontal Disease-The Role of Microvascular Dysfunction. Biology (Basel) 10(5). 10.3390/biology10050441 10.3390/biology10050441 PMC8156280 34067557

[10] Petschke FT, Engelhardt TO, Ulmer H, Piza-Katzer H (2006). [Effect of cigarette smoking on skin perfusion of the hand]. Chirurg.

[11] McDaniel JC, Browning KK (2014) Smoking, chronic wound healing, and implications for evidence-based practice. J Wound Ostomy Continence Nurs. ;41(5):415 – 23; quiz E1-2. 10.1097/WON.0000000000000057 10.1097/WON.0000000000000057 PMC4241583 25188797

[12] Lalla E (2007). Periodontal infections and diabetes mellitus: when will the puzzle be complete?. J Clin Periodontol.

[13] Yalda B, Offenbacher S, Collins JG (1994). Diabetes as a modifier of periodontal disease expression. Periodontol 2000.

[14] Goodson WH, Hunt TK (1978). Wound healing in experimental diabetes mellitus: importance of early insulin therapy. Surg Forum.

[15] Sharma S, Schaper N, Rayman G, Microangiopathy (2020). Is it relevant to wound healing in diabetic foot disease?. Diabetes Metab Res Rev.

[16] den Uil CA, Klijn E, Lagrand WK, Brugts JJ, Ince C, Spronk PE (2008). The microcirculation in health and critical disease. Prog Cardiovasc Dis.

[17] Kluz J, Malecki R, Adamiec R (2013). Practical importance and modern methods of the evaluation of skin microcirculation during chronic lower limb ischemia in patients with peripheral arterial occlusive disease and/or diabetes. Int Angiol.

[18] Ovadia-Blechman Z, Meilin A, Rabin N, Eldar M, Castel D (2015). Noninvasive monitoring of peripheral microcirculatory hemodynamics under varying degrees of hypoxia. Respir Physiol Neurobiol.

[19] Rejmstad P, Akesson G, Aneman O, Wardell K (2016). A laser doppler system for monitoring cerebral microcirculation: implementation and evaluation during neurosurgery. Med Biol Eng Comput.

[20] Spronk PE, Zandstra DF, Ince C (2004). Bench-to-bedside review: sepsis is a disease of the microcirculation. Crit Care.

[21] Barry O, Wang Y, Wahl G (2020). Determination of baseline alveolar mucosa perfusion parameters using laser Doppler flowmetry and tissue spectrophotometry in healthy adults. Acta Odontol Scand.

[22] Holzle F, Rau A, Loeffelbein DJ, Mucke T, Kesting MR, Wolff KD (2010). Results of monitoring fasciocutaneous, myocutaneous, osteocutaneous and perforator flaps: 4-year experience with 166 cases. Int J Oral Maxillofac Surg.

[23] Ciper N (2016) Kombinierte Gewebespektrometrie- Und laser-doppler-messungen bei parodontal erkrankten Zähnen sowie bei Abheilungsvorgängen Nach Extraktionen. Department of Orthodontics. University Hospital Bonn. https://nbn-resolving.org/urn:nbn:de:hbz:5n-42297

[24] Browne RH (1995). On the use of a pilot sample for sample size determination. Stat Med.

[25] Sim J, Lewis M (2012). The size of a pilot study for a clinical trial should be calculated in relation to considerations of precision and efficiency. J Clin Epidemiol.

[26] Beckert S, Witte MB, Konigsrainer A, Coerper S (2004). The impact of the Micro-lightguide O2C for the quantification of tissue ischemia in diabetic foot ulcers. Diabetes Care.

[27] Ishida H, Yamaguchi M, Saito SY, Furukawa T, Shannonhouse JL, Kim YS (2021). Na(+)-dependent inactivation of vascular na(+)/Ca(2+) exchanger responsible for reduced peripheral blood flow in neuropathic pain model. Eur J Pharmacol.

[28] Kreh A, Anton F, Gilly H, Handwerker HO (1984). Vascular reactions correlated with pain due to cold. Exp Neurol.

[29] Kolbenschlag J, Sogorski A, Kapalschinski N, Harati K, Lehnhardt M, Daigeler A (2016). Remote ischemic conditioning improves Blood Flow and Oxygen Saturation in Pedicled and Free Surgical flaps. Plast Reconstr Surg.

[30] Wang Y, Barry O, Wahl G, Chen B, Lin Y (2016). [Pilot study of laser-doppler flowmetry measurement of oral mucosa blood flow]. Beijing Da Xue Xue Bao Yi Xue Ban.

[31] Schreml S, Szeimies RM, Prantl L, Karrer S, Landthaler M, Babilas P (2010). Oxygen in acute and chronic wound healing. Br J Dermatol.

[32] Politis C, Schoenaers J, Jacobs R, Agbaje JO (2016). Wound healing problems in the Mouth. Front Physiol.

[33] Polimeni G, Xiropaidis AV, Wikesjo UM (2006). Biology and principles of periodontal wound healing/regeneration. Periodontol 2000.

[34] Shizukuishi S, Hanioka T, Tsunemitsu A (1988). Clinical application of tissue reflectance spectrophotometry to periodontal disease. Adv Dent Res.

[35] Grossi SG, Genco RJ, Machtei EE, Ho AW, Koch G, Dunford R (1995). Assessment of risk for periodontal disease. II. Risk indicators for alveolar bone loss. J Periodontol.

[36] Grossi SG, Zambon JJ, Ho AW, Koch G, Dunford RG, Machtei EE (1994). Assessment of risk for periodontal disease. I. Risk indicators for attachment loss. J Periodontol.

[37] Hanioka T, Tanaka M, Ojima M, Takaya K, Matsumori Y, Shizukuishi S (2000). Oxygen sufficiency in the gingiva of smokers and non-smokers with periodontal disease. J Periodontol.

[38] Buduneli N, Scott DA (2018). Tobacco-induced suppression of the vascular response to dental plaque. Mol Oral Microbiol.

[39] Mullally BH, Breen B, Linden GJ (1999). Smoking and patterns of bone loss in early-onset periodontitis. J Periodontol.

[40] Grudianov AI, Kemulariia IV (2010). [Laser doppler estimation of the influence of tobacco-smoking on the blood microcirculation in the periodont at the patients with the different stages of periodontal diseases]. Stomatologiia (Mosk).

[41] Bergström J, Boström L (2001). Tobacco smoking and periodontal hemorrhagic responsiveness. J Clin Periodontol.

[42] Genco RJ, Borgnakke WS (2013). Risk factors for periodontal disease. Periodontol 2000.

[43] Mirbod SM, Ahing SI, Pruthi VK (2001). Immunohistochemical study of vestibular gingival blood vessel density and internal circumference in smokers and non-smokers. J Periodontol.

[44] Taylor CT (2008). Interdependent roles for hypoxia inducible factor and nuclear factor-kappab in hypoxic inflammation. J Physiol.

[45] Eltzschig HK, Carmeliet P (2011). Hypoxia and inflammation. N Engl J Med.

[46] Baab DA, Oberg PA (1987). Laser Doppler measurement of gingival blood flow in dogs with increasing and decreasing inflammation. Arch Oral Biol.

[47] Matsuo M, Okudera T, Takahashi SS, Wada-Takahashi S, Maeda S, Iimura A (2017). Microcirculation alterations in experimentally induced gingivitis in dogs. Anat Sci Int.

[48] Preshaw PM (2009). Periodontal disease and diabetes. J Dent.

[49] Salvi GE, Yalda B, Collins JG, Jones BH, Smith FW, Arnold RR (1997). Inflammatory mediator response as a potential risk marker for periodontal diseases in insulin-dependent diabetes mellitus patients. J Periodontol.

[50] Auerswald S, Schreml S, Meier R, Blancke Soares A, Niyazi M, Marschner S (2019). Wound monitoring of pH and oxygen in patients after radiation therapy. Radiat Oncol.

[51] Rohleder NH, Flensberg S, Bauer F, Wagenpfeil S, Wales CJ, Koerdt S (2014). Can tissue spectrophotometry and laser doppler flowmetry help to identify patients at risk for wound healing disorders after neck dissection?. Oral Surg Oral Med Oral Pathol Oral Radiol.

[52] Donos N, D’Aiuto F, Retzepi M, Tonetti M (2005). Evaluation of gingival blood flow by the use of laser Doppler flowmetry following periodontal surgery. A pilot study. J Periodontal Res.

[53] Emshoff R, Moschen I, Oberrauch A, Gerhard S, Strobl H (2008). Outcomes of dental fracture injury as related to laser doppler flow measurements of pulpal blood-flow level. Dent Traumatol.

[54] Gleissner C, Kempski O, Peylo S, Glatzel JH, Willershausen B (2006). Local gingival blood flow at healthy and inflamed sites measured by laser Doppler flowmetry. J Periodontol.

[55] Hanioka T, Shizukuishi S, Tsunemitsu A (1990). Hemoglobin concentration and oxygen saturation of clinically healthy and inflamed gingiva in human subjects. J Periodontal Res.

[56] Retzepi M, Tonetti M, Donos N (2007). Gingival blood flow changes following periodontal access flap surgery using laser Doppler flowmetry. J Clin Periodontol.

[57] Sakr Y, Gath V, Oishi J, Klinzing S, Simon TP, Reinhart K (2010). Characterization of buccal microvascular response in patients with septic shock. Eur J Anaesthesiol.

[58] Forst T, Hohberg C, Tarakci E, Forst S, Kann P, Pfützner A (2008). Reliability of Lightguide Spectrophotometry (O2C®) for the investigation of skin tissue Microvascular Blood Flow and tissue oxygen supply in Diabetic and nondiabetic subjects. J Diabetes Sci Technol.

[59] Develioglu H, Kesim B, Tuncel A (2006). Evaluation of the marginal gingival health using laser Doppler flowmetry. Braz Dent J.

[60] Orekhova LY, Barmasheva AA (2013). Doppler flowmetry as a tool of predictive, preventive and personalised dentistry. EPMA J.

